# Zinc Finger Transcription Factor MZF1—A Specific Regulator of Cancer Invasion

**DOI:** 10.3390/cells9010223

**Published:** 2020-01-16

**Authors:** Ditte Marie Brix, Knut Kristoffer Bundgaard Clemmensen, Tuula Kallunki

**Affiliations:** 1 Cell Death and Metabolism, Center for Autophagy, Recycling and Disease, Danish Cancer Society Research Center, 2100 Copenhagen, Denmark; dittemariebrix@gmail.com (D.M.B.); knucle@cancer.dk (K.K.B.C.); 2 Danish Medicines Council, Dampfærgevej 27-29, 2100 Copenhagen, Denmark; 3 Department of Drug Design and Pharmacology, Faculty of Health Sciences, University of Copenhagen, 2200 Copenhagen, Denmark

**Keywords:** cancer therapy, EMT, lysosome, lysosome-mediated invasion, MZF1, phosphorylation, PAK4, SUMOylation, transcription factor, zinc finger

## Abstract

Over 90% of cancer deaths are due to cancer cells metastasizing into other organs. Invasion is a prerequisite for metastasis formation. Thus, inhibition of invasion can be an efficient way to prevent disease progression in these patients. This could be achieved by targeting the molecules regulating invasion. One of these is an oncogenic transcription factor, Myeloid Zinc Finger 1 (MZF1). Dysregulated transcription factors represent a unique, increasing group of drug targets that are responsible for aberrant gene expression in cancer and are important nodes driving cancer malignancy. Recent studies report of a central involvement of MZF1 in the invasion and metastasis of various solid cancers. In this review, we summarize the research on MZF1 in cancer including its function and role in lysosome-mediated invasion and in the expression of genes involved in epithelial to mesenchymal transition. We also discuss possible means to target it on the basis of the current knowledge of its function in cancer.

## 1. Transcription Factors as Drug Targets in Cancer

For a long time, steroid receptors, which are also known as ligand-activated transcription factors, have been the main group of transcription factors targeted in anti-cancer treatments. During recent years, other sequence-specific transcription factors have emerged as promising anti-cancer drug targets and consequently, transcription factors have lost their status of being “undruggable”. This is especially the case for zinc finger transcription factors, which is a large group of proteins with their own specific DNA binding sequences. A good example of a cancer drug that targets a transcription factor is thalidomide, an antiemetic drug from the 1950s that has been repurposed as a novel treatment against hematological malignancies and which functions by inactivating zinc finger transcription factors Ikarios (IKZF1) and Aiolos (IKZF3) through their destabilization [1,2]. Here we will summarize the recent literature on the role and function of another cancer-relevant zinc finger transcription factor, Myeloid Zinc Finger 1 (MZF1), and present reasoning for its potential targeting in cancer and discuss the possibilities of how to target it.

## 2. What Is MZF1?

### 2.1. MZF1 Is a Sequence-Specific, Oncogenic Transcription Factor Involved in Myeloid Differentiation

MZF1 is a member of the SCAN domain-containing zinc finger transcription factor (SCAN-ZFP) family, a subfamily of zinc finger proteins (ZFPs)[3]. SCAN-ZFPs represent a class of DNA-binding proteins, many of which are known to regulate transcription during different developmental processes. MZF1 was first isolated from the peripheral blood from a patient with chronic myeloid leukemia and was described as a novel zinc finger protein involved in transcriptional regulation of hematopoietic development [4]. A few years later it was shown to regulate the expression of hematopoiesis-specific genes influencing differentiation, proliferation and programmed cell death and its aberrant expression was found to result in the development of hematopoietic cancers [5,6]. During the last decade, MZF1 was shown to be implicated in the development of various types of solid cancers by enhancing cancer cell growth, migration and invasion [7,8,9,10,11,12,13,14,15]. Knowledge on the detailed mechanisms by which MZF1 activity is regulated and the central target genes it activates has steadily increased and is still emerging due to active research on the topic.

### 2.2. MZF1 Transcript Variants and Functional Domains

**Figure 1:**
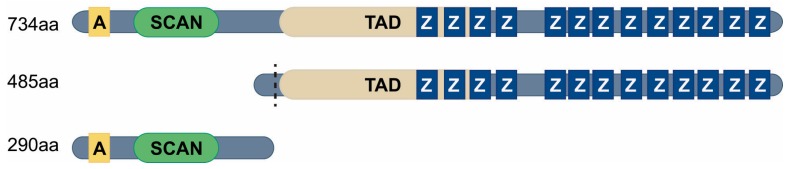
MZF1 protein isoforms. Top: Domain structure of the full-length (734 amino acid) MZF1 isoform containing five distinct domains: acidic domain (A), SCAN domain (SCAN), transactivation domain (TAD), and 13 highly conserved Krüppel-like zinc finger motifs (Z) arranged in two domains. Middle: Domain structure of the putative (485 amino acid) “zinc finger only”-form of MZF1, that in addition to 13 zinc fingers also has the TAD domain. The amino terminus of the new, recently identified 450 kD zinc finger only isoform is marked with a dashed black line. Bottom: Domain structure of the 290 amino acids “SCAN domain only” form of MZF1 that in addition to the SCAN domain also has the acidic domain (A).

MZF1 is encoded by a single-copy gene located at chromosome 19q13.4, which is the sub-telomeric region of the chromosome 19q, containing a large number zinc finger genes [4]. Full-length MZF1 protein is estimated to be about 82 kD without post translational modifications. *MZF1* gene supposedly encodes three transcript variants, which are predicted to result from alternative use of two transcription initiation sites and from alternative splicing [16,17]. MZF1 gene is composed of six exons [16,17]. Early work on *MZF1* transcripts lead to the identification of two human MZF1 isoforms: one full-length 734 amino acid isoform containing a SCAN domain in the N-terminus; 13 zinc finger DNA-binding domains in the C-terminus; and one N-terminally truncated, 485 amino acid isoform containing the 13 zinc finger DNA-binding domains and a short N-terminal fragment [16,17] (Figure 1).

Because the smaller 485 amino acid MZF1 isoform (the so-called zinc finger only isoform) was isolated and characterized first, it was for some years believed to be the full-length MZF1. Only when a novel mouse isoform of MZF1, then denoted as Mzf-2, was identified [16,18], was it discovered that also human MZF1 exists in a larger form, containing several additional structural and functional domains [17]. Soon after, these full-length MZF1 isoforms, Mzf-2a (mouse) and MZF1B/C (human), were collectively denoted as MZF1. A third MZF1 isoform of 290 amino acids containing only the SCAN domain in the N-terminus was later identified by the National Institutes of Health Mammalian Gene Collection Program (http://genecollections.nci.nih.gov/MGC/) (Figure 1). This so-called “SCAN domain only” isoform belongs to a group of proteins known as SCAND proteins. SCAND proteins are expected to function as regulators of other SCAN domain-containing proteins [19,20]. The tissue-specific expression and function of this isoform has not been elucidated. It also needs to be noted that the 485 amino acid “zinc finger only” isoform of MZF1 has been recently deleted from the human NBCI sequences and replaced with the predicted sequence of 450 amino acids “zinc finger only” form, where the N-terminus is slightly shorter than in the 485 amino acids form (Figure 1).

Full-length MZF1 consists of five distinctive domains (Figure 1). The domain furthest towards the N-terminus is called an acidic domain (A), which is rich in glutamic and aspartic acid [16,21]. This domain is involved in transcriptional activation and contains regulatory SUMO and phospho-sites [22]. It can also mediate interactions between MZF1 and other transcription factors [22,23,24]. Downstream of the acidic domain is the SCAN domain of 84 amino acids, a leucine-rich region known to mediate protein–protein interactions [16,17,25,26]. The SCAN domain is found in the SCAN-ZFP family of zinc finger proteins and it mediates homo- and heterodimer formation between SCAN domain containing zinc finger proteins [25,26,27,28]. Following the highly conserved SCAN domain there is a so-called transcriptional activation domain (TAD). It is a serine and threonine rich region that is involved in the transcriptional activation of MZF1 [18,21]. This MZF1 domain was identified as a TAD by a classical study by Ogawa and co-workers [21]. In the study, they showed that in murine MZF1 this domain is phosphorylated by mitogen-activated protein kinase ERK and p38 in three of its serines, serine 257, 275 and 295, leading to transcriptional inactivation of Mzf-2a. Consequently, substitution of all of these serines with alanines resulted in strong enhancement of its transcriptional activity in murine myeloid cell line LGM-1[21]. The corresponding sites are conserved in human MZF1, where they are represented as serine 256, 274 and 294. Later on, post-translational modifications in both the acidic domain and the SCAN domain were found to contribute to the transcriptional activity of human MZF1 [22]. In the C-terminal region of MZF1 are the 13 highly conserved Krüppel-like zinc finger motifs arranged in two domains. The first four zinc-finger motifs form one zinc-finger domain and the last nine motifs form another zinc-finger domain, separated from the first one by a glycine-proline-rich region of 24 amino acid residues [4] (Figure 1). The two zinc-finger domains of MZF1 bind to two distinct, yet similar DNA consensus sequences with a common core sequence of four or five guanines, which allows MZF1 to bind more than one DNA sequence at the same time [29], or to bind stronger in genomic sites containing binding sites for both motifs.

## 3. MZF1 and Cancer

### 3.1. MZF1 and Hematological Malignancies

MZF1 was originally isolated from chronic myeloid leukemia and was shown to be involved in hematopoietic differentiation due to its ability to control the expression of genes involved in hematopoiesis such as *CD34* and *MYB* [4,5,6]. Due to these reasons, most of the earliest studies on the function of MZF1 were done in hematopoietic cells. Some of the results concerning the actual role and function of MZF1 in hematopoietic malignancies are contradictory. This is because during the earliest studies of MZF1, the knowledge of MZF1 isoforms was not complete. Thus, many studies were done using overexpression of the so-called zinc-finger-only isoform of MZF1, that was the 485 amino acid isoform (Figure 1), which is practically missing most of the N-terminal regulatory domains. As mentioned, this zinc-finger-only isoform was later deleted from the human NBCI sequences, suggesting that it may be a cloning artifact. It has, however, been replaced with a slightly shorter, 450 amino acid isoform, which is a predicted alternative MZF1 transcript that can theoretically exist. In brief, early experiments involving overexpression of the 485 amino acid zinc-finger-only isoform in myeloid cells showed that it inhibits hematopoietic differentiation of mouse embryonic stem cells and delays retinoic acid-induced granulocytic differentiation and apoptosis by inducing proliferation in human promyeloblasts [6,30]. Contrary to what would be expected from these overexpression studies, silencing of MZF1 with antisense oligodeoxynucleotides (AOS) significantly inhibited granulocyte development in vitro from granulocyte colony-stimulating factor-induced cells originating from normal bone marrow, which was evident from granulocyte colony formation assays [31]. This result coincides with the results obtained from Mzf1 knockout mice, which accumulate highly proliferating myeloid cells in their bone marrow and liver, disturbing the tissue architecture, indicating that Mzf1 may function as a tumor suppressor in the hematopoietic compartment [32]. Since AOS and knockout studies target the full-length MZF1, it is understandable that the results obtained when downregulating or inhibiting MZF1 expression would not necessarily be the opposite of the results obtained when overexpressing the MZF1 zinc-finger-only isoform. The zinc-finger-only-isoform, by lacking the N-terminus that contains most of the regulatory domains of MZF1, would have impaired regulation of its activity and would be unable to dimerize with SCAN domain-containing proteins. However, it could mimic MZF1 in a potential state where it is void of its upstream regulation. This could be achieved for example via cancer-induced aberrant expression of MZF1. Thus, it can be concluded that aberrant expression and regulation of MZF1 can make it oncogenic, which is also supported by the fact that mouse embryonic fibroblasts that overexpress the zinc-finger-only MZF1 isoform form aggressive tumors in athymic mice and lose their contact inhibition and upregulate their growth ex vivo [33].

### 3.2. MZF1 Acts as an Oncogene in Solid Cancers

Several studies demonstrate that MZF1 can promote tumorigenesis of various solid cancers. These include breast, cervical, colorectal, liver, lung, and prostate cancer [7,8,9,11,12,13,14,15,34]. Many MZF1 target genes have a central role in cancer, and increased expression and/or activation of MZF1 induces cell growth, migration and invasion [7,8,9,10,11,12,13,14,15]. Below we will summarize most of the results obtained on MZF1 in some common solid cancers.

#### 3.2.1. MZF1 in Breast Cancer

MZF1 is needed for the invasion of ErbB2-expressing breast cancer cells [7]. In a study by Rafn and co-workers, ErbB2 activation was shown to induce the invasion of breast cancer cell spheroids via activation of a signaling network that involves TGFβ receptor 1 and 2 (*TGFBR1* and *2*), ERK2 (*MAPK1*), PAK4, PAK5 and PAK6 (*PAK4*, *PAK5* and *PAK6*), cdc42 binding protein kinase beta (*CDC42BPB*), and protein kinase Cα (PKCα; *PRKCA*). Activation of this signaling network leads to activation of MZF1 and MZF1-mediated induction of expression of lysosomal cysteine cathepsins B and L (*CTSB* and *CTSL1*). This work implied for the first-time involvement of lysosomes in the invasion of ErbB2-expressing cancer cells. It showed that MZF1, upon activation by ErbB2 signaling, can induce the pericellular accumulation of lysosomes at the invadosome-like cellular protrusions in invasive ErbB2 expressing breast cancer cells, thereby initiating and promoting their invasion [7]. Once lysosomes have travelled to the cell periphery, their hydrolytic content can be secreted into the extracellular space (lysosomal exocytosis). This can initiate and induce invasion mainly via cathepsin B, which cleaves and thereby activates matrix metalloproteases (MMP) 2 and 3 and the urokinase plasminogen activator [35,36,37]. Consistently, ErbB2-positive primary breast tumors exhibit increased mRNA and protein expression of cathepsins B and L. Supporting the in vivo connection of ErbB2 activation and cathepsins B and L, the positive correlation between ErbB2 and cathepsin B and L expression in invasive breast cancer was found to be significant [7].

Interestingly, MZF1 regulates the expression of TGFβ1 gene (*TGFB1*) in response to osteopontin-induced integrin signaling in human mesenchymal stem cells, where increased TGFβ signaling induces them to differentiate and adapt a cancer-associated fibroblast phenotype, a process that leads to increased tumor growth and metastasis [14]. TGFβ1 is considered as one of the main regulators of epithelial mesenchymal transition (EMT) [38] and ErbB2 overexpression is connected to TGFβ overexpression, secretion and activation of TGFβ signaling [39]. TGFβ signaling amplifies oncogenic ErbB2 signaling and promotes invasion and metastasis of ErbB2 positive cancer cells [40,41,42]. Since ErbB2-induced activation of MZF1 is enhanced by TGFβ receptor signaling and *TGFB1* is a MZF1 target gene, increased TGFβ signaling can further induce ErbB2 signaling via a feedback loop involving MZF1 activation. This may additionally lead to enhanced activation of other MZF1 target genes that are important for amplification of breast cancer signaling networks and promoting breast cancer cell migration and invasion, such as *PRKCA* [11]. Interestingly, a complex formation between Elk-1 and MZF1 has been shown to enhance *PRKCA* expression in a synergistic manner and its expression correlates positively with the expression of Elk1 and MZF1 in various breast cancer cell lines [11]. Moreover, a high level of MZF1 in triple-negative breast cancer cell lines Hs578T and MDA-MB-231 is associated with a mesenchymal phenotype with increased cell migration and invasion, which is mediated via insulin-like growth factor receptor (IGF1) [24]. Consequently, destabilization of MZF1 by the IGF1R-driven p38MAPK-Erα-SLUG-E-cadherin pathway leads to conversion of the invasion-promoting mesenchymal phenotype to the less invasive epithelial phenotype. In osteoblasts, which are involved in osteolytic breast cancer metastasis, MZF1 has been shown to upregulate the expression of another EMT regulator, N-cadherin (*CDH2*) [43]. Moreover, a MZF1 target gene *AXL*, which can be activated upon lapatinib resistance in ErbB2 positive breast cancer cells [44], has been shown to induce EMT in breast cancer cells [45]. This all suggests that MZF1 has a role in the development of aggressive breast cancer.

#### 3.2.2. MZF1 in Cervical and Colorectal Cancers

MZF1 activation has been implicated in the progression of cervical and colorectal cancer, where it increases invasion and metastasis, at least partially, via increased expression and activity of receptor tyrosine kinase AXL [8]. Increased expression of *AXL* has been connected to invasion and metastasis of many types of cancers [45,46,47]. Supportively, both MZF1 and AXL protein levels are considerably higher in colorectal tumors than in corresponding normal tissue [8]. MZF1 binds directly to the *AXL* promoter, leading to increased *AXL* mRNA and protein expression [8]. However, depletion of *AXL* by RNA interference only partially inhibits MZF1-induced migration and invasion of colorectal cancer cells, suggesting that additional MZF1-regulated genes are involved in this process. MZF1 is also central for the activation of the expression of Phosphoinositide -3-Kinase Regulatory Subunit 3 Gamma (*PIK3R3*), which is a regulatory subunit of PI3 kinase (PI3K) needed for PI3K signaling and is important for cancer cell proliferation [15]. In human papillomavirus infected cervical cancer, MZF1 induces the expression of another transcription factor, *NKX2-1*, which in turn upregulates a cancer stem cell regulator *FOXM1*, resulting in increased tumor growth and invasion [48]. In another study with SiHa human cervical cancer cells, MZF1 was shown to bind the matrix metalloprotease 2 (MMP2) promoter, and a bit surprisingly to suppress its expression, and thus was reported to function as a tumor suppressor in these cells [49].

#### 3.2.3. MZF1 in Liver and Lung Cancer

MZF1 regulates the expression of the PKCα gene, *PRKCA*, in human hepatocellular carcinoma cells, where it binds directly to the MZF1 binding site in the *PRKCA* promoter region [9,12]. Depletion of MZF1 with specific antisense oligonucleotides reduces proliferation, migration and invasion of hepatocellular carcinoma cells [9,12] and suppresses the growth of the corresponding xenografts [10,12]. In lung cancer, MZF1 activates the expression of the c-Myc gene (*MYC*) upon loss of the liver kinase B1 (*LKB1*) [13]. This results in enhanced migration and invasion of lung cancer cells and facilitates their growth in soft agar [13]. In tumors from lung adenocarcinoma patients there is a positive correlation between high MYC and MZF1 and low LKB1 expression. Importantly, lung adenocarcinoma patients with low *LKB1* expression have a shorter overall survival than patients with high *LKB1* expression [13]. In lung cancer, MZF1 can upregulate the expression of *NKX2-1*, which in turn increases the expression of *FOXM1* resulting in facilitated tumor growth and invasion [48]. On the other hand, in lung adenocarcinomas, the loss of *LKB1* is associated with *NKX2-1* expression [50].

#### 3.2.4. MZF1 in Prostate Cancer

The role of MZF1 in prostate cancer is somewhat more complicated. The expression of the cell division control 37 (*CDC37*) gene is increased in prostate cancer cells. Here, MZF1 was shown to bind to the promoter of *CDC37* and upregulate its expression [51]. As expected, depletion of MZF1 in prostate cancer cells decreases *CDC37* expression and reduces their tumorigenesis. Interestingly, SCAND1, a SCAN domain protein that can inhibit MZF1 by dimerizing with it, can upon overexpression accumulate at the MZF1 binding sites at the *CDC37* promoter and downregulate its expression-inhibiting tumorigenesis [51]. On the contrary, MZF1 was shown to have the opposite effect in PC3 and DU145 prostate cancer cells, where expression of MZF1 upregulated ferroportin (*FPN*), the only known mammalian iron exporter [52]. Depletion of MZF1 was found to decrease the expression of *FPN*, as expected, but in turn this was shown to result in enhanced cancer cell growth in addition to increased cytoplasmic iron retention [52]. Consequently, increase in the expression of MZF1 inhibited tumor growth, suggesting that in respect to *FPN* regulation in these prostate cancer cells, MZF1 can exhibit a tumor suppressor type of function.

#### 3.2.5. MZF1 in Other Type of Cancers

In glioma cell lines, MZF1 binds directly to the LIM-only protein 3 (*LMO3*) promoter and induces the expression of *LMO3* [53], which is a transcriptional co-activator that can act as an oncogene in glioma, one of the most aggressive and most common tumors of the central nervous system. *LMO3* is often overexpressed in gliomas and its expression correlates positively with poor prognosis [53]. The 19q chromosomal deletions together with the deletion of 1p are used to define the oligodendroglioma, which is a specific type of glioma with favorable prognosis and good response to chemotherapy [54]. Interestingly, the 19q chromosomal deletions in oligodendroglioma include the MZF1 locus as well as the locus of genes coding for many other zinc-finger proteins. In esophageal squamous cell carcinoma samples of 13 patients, MZF1 was found to be co-activated with three other transcription factors, SPIB, MAFG and NFE2L1 when compared to their paired non-cancerous tissues using microarray analysis, where the expression of 17 other transcription factors was suppressed [55]. In gastric cancer cells, MZF1 upregulates *MMP14* expression by directly binding to its promoter [56]. In the same study it was shown that in the clinical samples, *MZF1* expression correlated positively with *MMP14* expression in gastric cancer. On the contrary to this, another study where human gastric cancer samples were analyzed indicated that MZF1 expression was decreased during gastric cancer progression, which correlated with increased invasiveness of gastric cancer [57].

#### 3.2.6. Many MZF1 Target Genes Have a Role in Cancer

MZF1 exerts its activity via modulating the expression of its target genes. Aberrant MZF1 expression and activation results in transcriptional changes that increase cell growth, migration and invasion (see above and reviewed by Eguchi and co-workers [58]). In summary, MZF1 may promote invasion and migration partially by controlling the expression of kinases that are controlling these processes such as *AXL* and *PRKCA*. It can also increase expression of lysosomal, invasion-inducing and promoting hydrolases *CTSB* and *CTSL1*, which facilitate intra- and extracellular degradation of extracellular matrix components by their direct cleavage or by indirectly cleaving and activating matrix metalloproteases MMP2 and MMP3 and urokinase plasminogen activator, which in turn degrade the extracellular matrix [35,36]. MZF1 is also expected to have a role in EMT by controlling the expression of *TGFB1*, *CDH2* and *FOXM1*, and several other EMT-related genes. In Table 1, we have listed the known MZF1 target genes. However, it needs to be noticed that only the ones that are verified by chromatin immunoprecipitation can be considered definite direct targets of MZF1.

## 4. How Does MZF1 Function?

**Figure 2:**
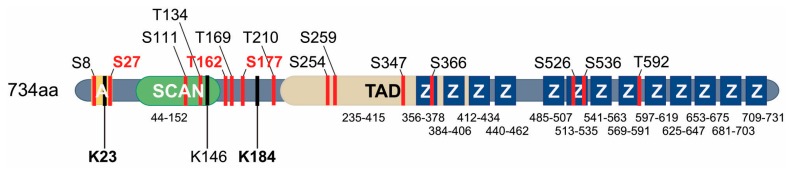
Schematic representation of the protein structure of full-length human MZF1 with reported SUMO-sites (K) and serine (S) and threonine (T) phosphorylation sites. The domain structure of MZF1 is presented as in Figure 1. The location of each indicated SUMO- and phospho-site is shown. The verified SUMO-sites are marked with bold font and the predicted SUMO-site (K146) is marked with regular font. The phospho-sites that are highlighted with red have been identified as ErbB2-responsive sites. Note that the serines 256, 274 and 294 corresponding to the ERK phosphorylation sites in the TAD of murine MZF1 have not yet been reported as phospho-sites in humans.

In order to target the oncogenic functions of MZF1, we need to understand how MZF1 is regulated. The key to MZF1 function in cancer lies in its domain structure and in its post-translational modifications (Figure 2) that are regulating its association with other factors, its activation status and its availability.

### 4.1. Regulation of MZF1 Expression

Relatively little is known about the transcriptional regulation of *MZF1*. Originally, MZF1 was identified as an important transcriptional regulator of myeloid differentiation, and its expression was believed to be myeloid-specific [76]. Later on, it was identified as an oncogenic transcription factor responsible for migratory and invasive phenotypes of various cancer cell lines. Analysis of TCGA website data indicates that there is a significant amplification of the *MZF1* gene in various solid cancers [58]. These include breast, bladder, lung, and uterine cancers. Indeed, increased expression of MZF1 protein levels has been detected in the study of 321 tissue microarray samples containing primary breast cancer and normal breast samples [77]. In these samples, MZF1 levels were shown to significantly increase from normal breast tissue to grade 1–2 tumors, which define invasive ductal carcinoma.

Non-coding RNAs have arisen as important regulators of gene expression. Several microRNAs (miRNAs) can regulate *MZF1* expression. Let-7 miRNAs belong to the group of miRNAs whose aberrant expression is most frequently associated with cancer [78]. Let-7 is upregulated during differentiation, and its expression is systematically downregulated in malignant cancers including breast cancer [79]. Let-7 binds to the 3′-untranslated region of *MZF1*, and ectopic expression of let-7 microRNAs let-7d and let-7e can efficiently downregulate *MZF1* and invasion of constitutively active ErbB2-expressing breast cancer cells [77]. MiR-492 is another microRNA that can bind to the 3′-untranslated region of *MZF1* [52]. MiR-492 regulates *MZF1* expression in prostate cancer cells, and in prostate tumors, miR-492 levels correlate reversibly with the levels of MZF1. Another study with glioma cell lines indicates that overexpression of miR-101 leads to a decrease in *MZF1* expression, without going further into detail in regard to its potential binding sites in *MZF1* [53]. MiRNA-337-3p inhibits gastric cancer progression by downregulating MZF1 activity via a specific mechanism, where miRNA-337-3p binds to the promoter region of *MMP14* adjacent to its MZF1 binding site and represses the MZF1-induced expression of *MMP14* [56]. Consequently, in the same study, miRNA-337-3p was shown to inhibit growth, invasion, metastasis, and angiogenesis of gastric cancer cells in vitro and in vivo via repression of MZF1 activity. Furthermore, miRNA-337-3p expression was found to be an independent prognostic factor for a favorable outcome in gastric cancer.

Interestingly, according to UCSC genome browser (https://genome.ucsc.edu), a validated long non-coding RNA exists that contains the whole MZF1 coding sequence resulting in 15,573 base pair antisense RNA (LOC100131691). Thus far, no studies exist of its actual regulation, expression or function, although it is tempting to speculate that it can have a regulatory role in the expression of MZF1.

### 4.2. Regulation of the Transcriptional Activity of MZF1

#### 4.2.1. Interaction with Other Transcription Factors

MZF1 has to dimerize to function as a transcription factor. MZF1 utilizes its SCAN domain to form homo- and heterodimers with other SCAN-domain transcription factors [25,26,27,28]. The possibility of heterodimerization via the SCAN domain exposes MZF1 to an additional level of regulation, since depending on its dimerization partner, MZF1 may function as a transcriptional activator or repressor. Known SCAN domain-containing MZF1 dimerization partners include SCAN-ZFP family members RAZ1, ZNF24, ZNF174, and ZNF202 [3,26,80], which are all heterodimerizing with MZF1 via a SCAN–SCAN interaction. A recent computational study has tried to shed new light on MZF1 SCAN domain interactions by identification and analysis of cancer-specific mutations in the MZF1 SCAN domain [81]. In this study, 23 cancer-specific mutations were identified in the MZF1 SCAN domain, which could affect MZF1 function by changing its dimerization capacity directly or indirectly via gain or loss of possible post-translational modifications (Nygaard et al., 2016). This work identified cysteine 69 as a potential regulator of MZF1 SCAN–SCAN interactions. Moreover, simultaneous expression and appearance of other SCAN and SCAND domain-containing proteins and possible cancer-inducing mutations in them could also affect MZF1 function for example by directly or indirectly replacing the binding partners of MZF1. However, this type of exiting regulation scheme is still mostly theoretical, especially in the case of MZF1 heterodimers, since detailed biological information on the specific regulation of the transcriptional activity of MZF1 via heterodimeric SCAN–SCAN interactions is still missing.

In addition to SCAN-domain proteins, MZF1 can interact with proteins without the classical SCAN domain, which complicates the scenario of its regulation by binding partners. Moreover, MZF1 interaction with other proteins can even occur via other domains than the SCAN domain. Recent work has indicated that the acidic domain of MZF1 is an additional protein–protein interaction domain. The acidic domain of MZF1 is involved in its association with Elk1 in triple negative breast cancer [12,23,24]. Association of MZF1 and Elk1 via the acidic domain of MZF1 and the heparin-binding domain of Elk1 increases invasion, migration and mesenchymal phenotype of breast cancer cells. This occurs via increasing the expression of *PRKCA* and *IGF1R* by direct binding of MZF1 to their promoter regions. In non-invasive MCF7 breast cancer cells, MZF1 interacts with the CCCTC-binding factor (CTCF) via its acidic domain, which results in downregulation of the transcriptional activity of MZF1 [22]. Activation of ectopic ErbB2 signaling results in SUMO-directed (SUMOylation of lysine 23) phosphorylation of MZF1 serine 27 at its acidic domain, which dissociates MZF1 from its transcriptional repressor CTCF, allowing transcriptional activation of MZF1 [22].

#### 4.2.2. SUMOylation of MZF1

The activity of transcription factors can be modulated by covalent attachment of small ubiquitin-related modifier (SUMO) proteins: SUMO1, SUMO2, SUMO3, and SUMO4 in their SUMO acceptor sites [82]. According to current knowledge of consensus SUMOylation sites, MZF1 has three predicted SUMOylation sites: lysine 23, 184 and 146 (Figure 2) [22,58]. An earlier study that was the first to report MZF1 SUMOylation, suggested that a SUMOylation site would reside in the amino terminus of MZF1 between amino acids 15–27 [80], which is a conserved sequence found in a subset of SCAN-ZFPs [19,80]. This site was identified by showing that overexpressed full-length MZF1 has the ability to accumulate into promyelocytic leukemia nuclear bodies (PML-NBs), a function which requires SUMOylation, and which could be abolished when this area was deleted from MZF1 [80].

SUMOylation of transcription factors and their cofactors may lead to transcriptional activation or inactivation [82,83]. Several studies suggest an important role of PML-NBs in transcriptional regulation [84,85]. Especially, PML-NBs has been presented as a site where SUMO-conjugation occurs and where SUMOylated nuclear proteins reside and accumulate [86]. PML-NBs usually associate to the areas of genomic regions with high transcriptional activity, and many transcription factors can be transiently recruited to PML-NBs. Since SUMOylation usually occurs in the nucleus [83], introduction of mutations in the nuclear SUMO-modified proteins that interfere with their SUMOylation can promote their translocation into the cytoplasm. Thus, MZF1 SUMOylation may be involved in its nuclear retention and its ability to function as a transcription factor.

MZF1 lysine 23 has been identified as a SUMOylation site that directly regulates the transcriptional activity of MZF1 [22]. Its occupation by SUMO groups exposes a nearby serine 27 for phosphorylation by PAK4 in response to ErbB2 activation, which in turn results in increased transcriptional activity of MZF1, indicating its importance for the transcriptional activation of MZF1 as well as defining a new mechanistic type of post-translational regulation, “SUMO-directed phosphorylation”. In the same study, lysine 184 was mapped as an additional functional SUMO acceptor site. However, no biological function has yet been identified for it. It is tempting to speculate that MZF1 SUMOylation may be regulating its stability. However, in our studies of MZF1 post-translational modification and their effect on protein stability, we found that in ErbB2-expressing breast cancer cells, MZF1 is a very stable protein and its stability was not affected by mutating its SUMO sites (Brix and Kallunki, unpublished observations). Moreover, in the ErbB2-expressing breast cancer cells, no evidence of SUMOylation of lysine 146 was found [22]. It is possible that lysine 146 is not a functional SUMO acceptor site in MZF1. Supporting this, its probability as a SUMO acceptor site is much lower than that of lysine 23 and 184 [22,58].

#### 4.2.3. Phosphorylation of MZF1

**Figure 3:**
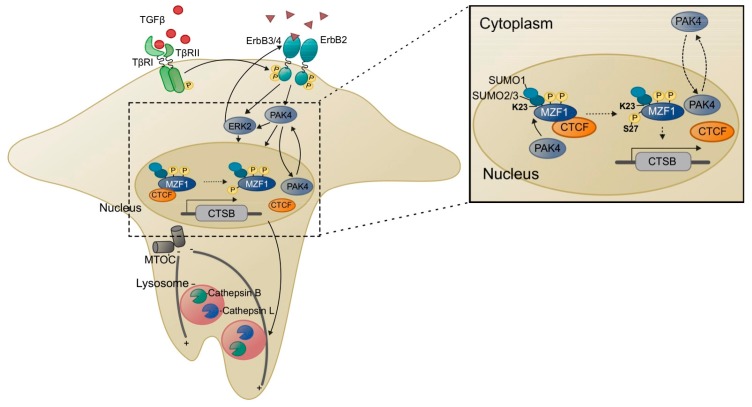
Graphical presentation of MZF1 activation and induction of *CTSB* expression in lysosome-mediated invasion as a response to ErbB2 signaling. ErbB2 activation, further supported by activation of TGFβ signaling, activates ERK2 and PAK4. Active PAK4 will phosphorylate MZF1 serine 27, if its adjacent lysine 23 is SUMOylated, which exposes MZF1 serine 27 to PAK4 phosphorylation. As a response to phosphorylation of serine 27, MZF1 association of its transcriptional repressors, e.g., CTCF, is prevented and MZF1 can now activate *CTSB* expression and lysosome redistribution, which leads to lysosome-mediated invasion.

MZF1 is a phosphoprotein that contains several potential as well as functional phosphorylation sites. Even though its massive phosphorylation has been known for some time, thanks to the large amount of phosphorylation analysis done by Mass Spectrometry that has been deposited on the web (http://www.phosphosite.org), thus far no biological function has been shown for the majority of these sites. In a study conducted in MCF7 breast cancer cells with inducible expression of constitutively active ErbB2, ErbB2 activation was shown to increase the transcriptional activity of MZF1 via a signaling network that involves TGFβ receptors 1 and 2 (TGFBR1 and 2), ERK2, PAK4 (PAK5 and PAK6), cdc42 binding protein beta kinase (CDC42BPB), and PKCα (PRKCA) [7]. In a recent study that utilized ErbB2 positive breast cancer cells for phosphorylation analysis by Mass Spectrometry, 13 MZF1 phosphorylation sites were identified [22]. Only three of these were phosphorylated in response to ErbB2 activation. These were serine 27, serine 162 and threonine 177, other sites being constitutively phosphorylated. Of these three sites, only phosphorylation of serine 27 was shown to increase the transcriptional activity of MZF1, the two others having no significant effect on it [22]. Supporting the importance of this phospho-site in vivo, serine 27 phosphorylation was found in an ErbB2-positive breast tumor sample in a proteomics study covering 105 breast tumors that were characterized for TCGA [87]. Furthermore, MZF1 serine 27 phosphorylation was found to correlate positively and significantly with ErbB2 status in a breast tumor tissue microarray containing 225 tissue cores embedded as duplicates and enriched with primary invasive breast cancer samples [22]. The phosphorylation of serine 27 is tightly connected to the SUMOylation of lysine 23 through a mechanism where SUMOylation of lysine 23 is needed as a prerequisite for the phosphorylation of serine 27 by PAK4. In silico modelling of this activation mechanism suggests that SUMOylation of lysine 23 opens up and exposes the serine 27, which otherwise is masked and not approachable for PAK4 to dock to it and phosphorylate it (Figure 3). Phosphorylation of serine 27 will then dissociate MZF1 from the transcriptional repressor CTCF, allowing MZF1 to activate transcription of *CTSB* needed for the invasion of ErbB2-expressing cells. Interestingly, a recent study in human esophageal cancer cell lines demonstrated that phosphorylation of MZF1 serine 27 by constitutively active casein kinase 2 (CK2), which is often upregulated in cancers, mediates their epithelial to mesenchymal transition by inducing the expression of N-cadherin [60].

#### 4.2.4. Mutations Creating New MZF1 Binding Sites in the Genome

Cancer-induced somatic mutations can create new transcription factor binding sites at the regulatory regions of cancer genes [88]. Mutations at the promoter regions of genes that are important for cancer progression can create new transcription factor binding sites that can contribute to the overexpression of that particular gene. Tian and co-workers have identified a mechanism by which MZF1 can affect gene expression via cancer-induced allelic mutations that result in a novel transcription factor co-operation at the promoter of the hepatocyte growth factor gene *HGF* [89]. They have identified single-nucleotide polyformism (SNP) and single nucleotide variants (SNV) in multiple myeloma at the promoter region of *HGF* that result in its increased expression. These mutations resemble the wild-type sequences of the binding motifs of MZF1, nuclear factor kappa-B (NFκB) and nuclear factor erythroid 2-related factor 2 (NFR-2), which together can contribute to increased expression of *HGF*. Whether this is a single type of cancer and a gene where new MZF1 binding sites are gained through a cancer-induced mutation or if multiple cancers and promoters are involved, is not yet known.

## 5. How Does MZF1 Promote Cancer Invasion and Metastasis?

The mechanistical explanations of how MZF1 promotes cancer progression must rely on the activation of its specific target genes in cancer. Currently known MZF1 target genes have been mapped in individual functional studies, however, no genome-wide studies on MZF1 transcriptional targets have been reported. The majority of the known MZF1 target genes are known cancer genes, whose activation is expected to promote cancer progression (Table 1). Two of the invasive processes activated by MZF1 have been described in more detail. We will briefly present these below.

### 5.1. Lysosomes, MZF1 and Invasion

**Figure 4:**
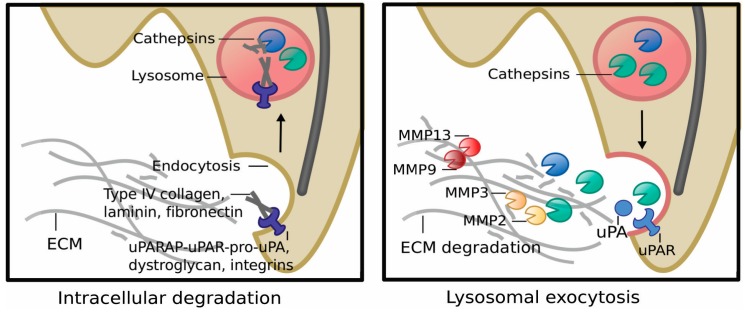
Graphical presentation of cellular mechanisms activated in lysosome-mediated invasion. Peripheral lysosomes contribute to extracellular matrix (ECM) degradation both internally (**left**) and externally (**right**). Peripheral lysosomes degrade the ECM components that have been internalized by the cell e.g., via endocytosis. Peripheral lysosomes can secrete their contents, including cathepsin B, into the extracellular space via lysosomal exocytosis, a process where the lysosome membrane fuses with the plasma membrane, which allows the secretion of the lysosomal contents to the extracellular space. Secreted cathepsin B degrades the ECM components: type IV collagen, laminin and fibronectin and initiates the activation of the extracellular degradome by cleaving the pro-forms of urokinase plasminogen activator and MMP2 and MMP3, which are activators of MMP9 and MMP13.

Lysosomes have a central role in the induction of invasion by ErbB2 in breast cancer cells [90]. Invasion of the MCF7 breast cancer spheroids expressing the trastuzumab-resistant p95 form of ErbB2 depends on the activation of a signaling network that culminates in the activation of MZF1 [7]. Here MZF1 regulates the function and activity of lysosomes by mediating ErbB2-induced, increased expression of lysosomal cysteine cathepsins B and L (*CTSB* and *CTSL1*), which is necessary for the invasion of these cells. Increased expression of *CTSB* and *CTSL1* leads to increased activity of cathepsins B and L, whose expression correlates positively (*p* < 0.0001) with high ErbB2 status in primary invasive breast cancer [7]. This is connected to the redistribution of lysosomes from a perinuclear to a peripheral position in invadosome-like cellular protrusions adjacent to the cell membrane, which is induced by phosphorylation of MZF1 serine 27 [22]. The appearance of the peripheral population of lysosomes correlates positively with the invasiveness of ErbB2 positive ovarian and breast cancer cells [7,91] and can contribute to extracellular matrix (ECM) degradation both internally and externally [34,36,37]. Peripheral lysosomes degrade the ECM components that have been internalized by the cell. Moreover, they can secrete their hydrolytic content, including cathepsin B, into the extracellular space to initiate and promote invasion. Secreted cathepsin B degrades the ECM components type IV collagen, laminin, and fibronectin and initiates the activation of the extracellular degradome by cleaving the pro-forms of urokinase plasminogen activator and MMP2 and MMP3 [92], which are activators of MMP9 and MMP13 (Figure 4). MZF1 seems to be a central regulator of invasion-associated pericellular lysosome distribution and lysosome-mediated invasion of ErbB2 expressing highly invasive cancer cells [7,22,90] (Figure 3).

### 5.2. MZF1 and EMT

Recently, MZF1 has been connected to EMT, a biological process where epithelial cells lose their polarity and cell–cell adhesion capability and gain invasive and migratory properties by adapting a mesenchymal phenotype. In human esophageal cancer cell lines, phosphorylation of MZF1 serine 27 by CK2 initiates EMT by inducing the transcription of N-cadherin during the EMT-inducing switch from E-cadherin to N-cadherin [60]. Knockdown of MZF1 by specific shRNA reverses the mesenchymal phenotype of these cells into epithelial and downregulates the expression of N-cadherin. In triple-negative breast cancer cells, MZF1 activation can maintain the mesenchymal phenotype by interacting with Elk1 at the promoter region of *IGF1R* [24]. Even though evidence of the connection between MZF1 and EMT is increasing, it is still not clear if the role of MZF1 in EMT is cancer type-specific, or if MZF1 can have a more general role in the initiation and/or maintenance of EMT. Intriguingly, 17 of the 31 (55%) reported MZF1 target genes (Table 1) are somehow involved in EMT in other cancer studies.

## 6. Conclusions and Future Directions

The majority of studies on MZF1 in cancer report that MZF1 functions as an oncogene in various solid cancers by regulating the expression of genes involved in cancer progression, EMT, extracellular matrix degradation, invasion, and angiogenesis. Inhibition of MZF1 function could be a way to inhibit these processes. Different efficient approaches to inhibit transcription factor activity in cancer exist, including transcription factor destabilization by affecting the post-translational modifications that regulate stability or activity. Regarding MZF1, probably one of the most successful scenarios could be to inhibit its association with its specific co-transcription factors such as Elk1, which is needed for MZF1-induced activation of the expression of *PRKCA* and *IGF1R*, and which contributes to the stability of MZF1 in triple-negative breast cancer [24].

Specific post-translational modifications of MZF1 are induced in invasive cancer, as is the case for breast cancer harboring ErbB2 activation, and these are necessary for the invasive signaling mediated via MZF1 in response to ErbB2 activation in breast cancer cells [22]. Thus, another valid possibility would be to target the enzymes responsible for these post-translational modifications, namely SUMOylation of lysine 23 and/or phosphorylation of serine 27. It is not known what regulates the SUMOylation of lysine 23, which is a prerequisite of the phosphorylation of serine 27 by PAK4 [22]. However, a theory exists according to which the generally high phosphorylation status of MZF1, and especially the phosphorylation of serine 8 can bend the MZF1 molecule to a position where lysine 23 is exposed to SUMOylation [22,81]. Interestingly, increased SUMOylation is generally connected to cancer progression, and in breast cancer it is associated with poor prognosis [93,94]. The expression of SUMOylation-associated enzymes is often increased in cancer, and thus, numerous SUMO-pathway-targeting inhibitors have been developed, many of which can be considered as promising anti-cancer agents [95]. These could also target SUMOylation of MZF1 lysine 23 and thus prevent the activation of MZF1 by hindering the phosphorylation of serine 27.

PAK4, a kinase that can phosphorylate MZF1 serine 27 in response to ErbB2 activation, is considered as a good target for the treatment of a variety of solid cancers including breast cancer, and its inhibition for this purpose has been patented by Hoffman-La Roche and Genentech [96]. Although the resulting PAK4 inhibitor PF-3758309 failed in phase I clinical trials [97], a new PAK4 inhibitor, KPT-9274, has been developed (Karyopharm Therapeutics, USA), which is currently in phase I clinical trials [98]. Identification of MZF1 as an oncogenic target of PAK4, whose activity is important for invasiveness of ErbB2 positive breast cancer cells, suggests that PAK4 inhibitors might be useful for the treatment of cancers whose aggressiveness depends on MZF1.

Another possible way to target MZF1 could be by preventing its binding to the regulatory regions of its cancerous target genes. For this, more understanding of its DNA-binding specificity would be needed. For example, since it has two distinct zinc finger domains with divergent binding sequences, it would be useful to find out if either of them is a preferred binding domain for its target genes that are important in cancer. Interestingly, by using CRISPR-Cas9 gene editing technology, we have experienced that ErbB2-expressing breast cancer cell lines have developed dependency on *MZF1*, so that these cancer cells harboring full knockout of *MZF1* are not viable [22], suggesting that they could have developed oncogene addiction towards MZF1. If this is the case, efficient inhibition of MZF1 could result not only in inhibition of invasion but could also be lethal for them.

An increasing number of studies point to a central role for enhanced MZF1 expression and activation in the invasiveness of different solid cancers, making it an attractive therapeutic target. Several probabilities already exist for how its activity could be controlled, and at the same, interesting possibilities still remain to be studied. One potentially useful future approach would be to carry out an in silico screen to identify compounds that interfere with MZF1 DNA binding, dimerization with specific partners or with post-translational modifications that are important for its activation. Especially, its DNA binding domain as well as SUMOylation of lysine 23 and phosphorylation of serine 27 are well characterized. These modification sites are located in domains for which crystal structures are available and would thus already be suitable for such an approach. To use this approach to identify molecules that can prevent MZF1 heterodimerization, more research would be needed to understand which associations are beneficial for cancer. In general, more research is still needed to increase the understanding of the detailed function of MZF1 in cancer, of the cellular cancer-promoting programs it regulates, the cancers where its inhibition would be most beneficial, and how it should be achieved.

## Figures and Tables

**Table 1:** MZF1 target genes; their method of identification (ChIP: chromatin immunoprecipitation; EMSA: electrophoretic mobility shift assay), reference, function (the role of MZF1) and their involvement generally in EMT (yes, if involvement has been reported).

Gene	Method	Reference	Function	EMT
AXL	ChIP, Luciferase	[8]	Activator	yes
CD14	EMSA,luciferase	[59]	Activator	yes
CD34	EMSA, Acetyltransferase activity	[6]	Activator	
CDC37	ChIP, Luciferase	[51]	Activator	
CDH2 (N-Cadherin)	EMSA	[43]	Activator	yes
CDH2 (N-Cadherin)	ChIP, Luciferase	[60]	Activator	
CK17	Luciferase, qPCR	[61]	Activator	
CTGF	ChIP	[62]	Activator	yes
CTSB	ChIP, Luciferase	[7]	Activator	
GAPDH	ChIP	[63]	Activator	yes
HK2	ChIP	[64]	Suppressor	
IGFIR	ChIP, Luciferase	[65]	Suppressor	yes
ITGAM (CD11b)	EMSA, luciferase	[59]	Activator	
LMO3	ChIP	[53]	Activator	
MMP2	ChIP, Luciferase	[49]	Suppressor	yes
Mtor	ChIP, EMSA, Luciferase	[66]	Suppressor	yes
MYB (c-myb)	EMSA, Acetyltransferase activity	[6]	Activator	
MYC	ChIP, Luciferase	[13]	Activator	
NFKB1A	ChIP	[67]	Activator	yes
NKX2-1	ChIP, Luciferase	[48]	Activator	yes
NKX2-5	ChIP, Luciferase	[68]	Activator	yes
NOV	ChIP	[61]	Activator	
OOCT4	Luciferase	[69]	Activator	yes
PAX2	Luciferase	[70]	Suppressor	yes
PIK3R3 (p55PIK)	ChIP, Luciferase	[15]	Activator	yes
PRAME	ChIP	[71]	Activator	yes
PRKCA (PKC alpha)	ChIP, Luciferase	[12]	Activator	
SLC40A1 (FPN)	ChIP, Luciferase	[52]	Activator	
SMAD4	ChIP, EMSA, Luciferase	[72]	Activator	
TGFB1	ChIP, Luciferase	[73]	Activator	yes
TNFRSF10B (DR5)	Luciferase	[74]	Activator	
YAP1	ChIP, EMSA, Luciferase	[75]	Activator	yes
